# Retraction: Integrated use of phosphorus, farmyard manure and biofertilizer improves the yield and phosphorus uptake of black gram in silt loam soil

**DOI:** 10.1371/journal.pone.0273465

**Published:** 2022-08-31

**Authors:** The PLOS ONE Editors

The *PLOS ONE* Editors retract this article [1] because it was identified as one of a series of submissions for which we have concerns about authorship, competing interests, and peer review. We regret that the issues were not addressed prior to the article’s publication.

JS, RB, BSD, MHS, HMA, and RK did not agree with the retraction. DSS, AAAH, AA, and FK either did not respond directly or could not be reached.
